# Supplementary material for: A single Spanish version of maternal and paternal postnatal attachment scales: validation and conceptual analysis
Source: PeerJ. 2018 Nov 30;6:e5980. doi: 10.7717/peerj.5980 (PMC6276590; doi:10.7717/peerj.5980)
Supplement: Supplemental Information 7 [file peerj-06-5980-s007.pdf]

SPANISH VERSION OF MATERNAL POSTNATAL ATTACHMENT SCALE (MPAS)

En las siguientes preguntas, por favor elije la opción que mejor describa tus sentimientos durante el cuidado del bebé.

1. Cuando estoy cuidando al bebé, me siento fastidiada o irritada:

☐ Muy frecuentemente.

☐ Frecuentemente.

☐ Ocasionalmente.

☐ Muy raramente.

☐ Nunca.

2. Cuando estoy cuidando al bebé siento que se está portando mal a propósito o intentando fastidiarme:

☐ Muy frecuentemente.

☐ Frecuentemente.

☐ Ocasionalmente.

☐ Muy raramente.

☐ Nunca.

3. En las últimas dos semanas, podría describir mis sentimientos hacia el bebé como:

☐ Desagrado.

☐ Falta de sentimientos intensos hacia el bebé.

☐ Ligero cariño.

☐ Cariño moderado.

☐ Cariño intenso.

4. Respecto a mi relación con el bebé, en general:

☐ Me siento muy culpable por no implicarme más.

☐ Me siento bastante culpable por no implicarme más.

☐ Me siento moderadamente culpable por no implicarme más.

☐ Me siento ligeramente culpable por no implicarme más.

☐ No me siento nada culpable por no implicarme más.

5. Cuando interactúo con el bebé me siento:

☐ Muy competente y segura.

☐ Bastante competente y segura.

☐ Moderadamente competente y segura.

☐ Ligeramente competente y segura.

☐ Nada competente y segura.

6. Cuando estoy con el bebé me siento tensa y nerviosa:

☐ Muy frecuentemente.

☐ Frecuentemente.

☐ Ocasionalmente.

☐ Muy raramente.

☐ Nunca.

7. Cuando estoy con el bebé y otras personas están presentes, me siento orgullosa de él/ella:

☐ Muy frecuentemente.

☐ Frecuentemente.

☐ Ocasionalmente.

☐ Muy raramente.

☐ Nunca.

8. Intento implicarme jugando con el bebé tanto como me es posible:

☐ Muy frecuentemente.

☐ Frecuentemente.

☐ Ocasionalmente.

☐ Muy raramente.

☐ Nunca.

9. Cuando no tengo más remedio que dejar al bebé:

☐ Me cuesta mucho irme.

☐ Me cuesta bastante irme.

☐ Me cuesta algo irme.

☐ Me cuesta poco irme.

☐ No me cuesta nada irme.

10. Cuando estoy con el bebé disfruto:

☐ Muy frecuentemente.

☐ Frecuentemente.

☐ Ocasionalmente.

☐ Muy raramente.

☐ Nunca.

11. Cuando no estoy con el bebé, me doy cuenta de que pienso en él/ella:

☐ Casi todo el tiempo.

☐ Muy frecuentemente.

☐ Frecuentemente.

☐ Ocasionalmente.

☐ Nunca.

12. Cuando estoy con el bebé intento alargar el tiempo que estoy con él/ella:

☐ Muy frecuentemente.

☐ Frecuentemente.

☐ Ocasionalmente.

☐ Muy raramente.

☐ Nunca.

13. Cuando he estado un tiempo separada del bebé y estoy a punto de encontrarme con él/ella de nuevo, normalmente siento:

☐ Placer intenso ante la idea.

☐ Placer moderado ante la idea.

☐ Leve placer ante la idea.

☐ Ningún sentimiento ante la idea.

☐ Sentimientos negativos ante la idea.

14. En este momento pienso en el bebé como algo propio, como algo mío:

☐ Completamente como MI bebé.

☐ Bastante como MI bebé.

☐ Algo como MI bebé.

☐ Un poco como MI bebé.

☐ Aún no lo siento como MI bebé.

15. Respecto a las cosas que hemos tenido que dejar por causa del bebé:

☐ Me arrepiento completamente.

☐ Me arrepiento bastante.

☐ Me arrepiento algo.

☐ Me arrepiento poco.

☐ No me arrepiento para nada.

16. En los últimos 3 meses, he sentido que no tenía tiempo para mí misma o para hacer las cosas que me interesan:

☐ Muy frecuentemente.

☐ Frecuentemente.

☐ Ocasionalmente.

☐ Muy raramente.

☐ Nunca.

17. Cuidar del bebé es una gran carga de responsabilidad. Estoy:

☐ Completamente de acuerdo con esto.

☐ Bastante de acuerdo con esto.

☐ Algo de acuerdo con esto.

☐ Muy poco de acuerdo con esto.

☐ Nada de acuerdo con esto.

18. Confío en mi propio criterio para decidir lo que el bebé necesita:

☐ Muy frecuentemente.

☐ Frecuentemente.

☐ Ocasionalmente.

☐ Muy raramente.

☐ Nunca.

19. Normalmente cuando estoy con el bebé:

☐ Soy muy impaciente.

☐ Soy bastante impaciente.

☐ Soy moderadamente impaciente.

☐ Soy ligeramente impaciente.

☐ No soy nada impaciente.
