# Supplementary material for: A single Spanish version of maternal and paternal postnatal attachment scales: validation and conceptual analysis
Source: PeerJ. 2018 Nov 30;6:e5980. doi: 10.7717/peerj.5980 (PMC6276590; doi:10.7717/peerj.5980)
Supplement: Supplemental Information 8 [file peerj-06-5980-s008.pdf]

SPANISH VERSION OF PATERNAL POSTNATAL ATTACHMENT SCALE (PPAS)

En las siguientes preguntas, por favor elije la opción que mejor describa tus sentimientos durante el cuidado del bebé.

1. Cuando estoy cuidando al bebé, me siento fastidiado o irritado:

☐ Muy frecuentemente.

☐ Frecuentemente.

☐ Ocasionalmente.

☐ Muy raramente.

☐ Nunca.

2. Cuando estoy cuidando al bebé siento que se está portando mal a propósito o intentando fastidiarme:

☐ Muy frecuentemente.

☐ Frecuentemente.

☐ Ocasionalmente.

☐ Muy raramente.

☐ Nunca.

3. En las últimas dos semanas, podría describir mis sentimientos hacia el bebé como:

☐ Desagrado.

☐ Falta de sentimientos intensos hacia el bebé.

☐ Ligero cariño.

☐ Cariño moderado.

☐ Cariño intenso.

4. Sé lo que mi bebé necesita o quiere:

☐ Muy frecuentemente.

☐ Frecuentemente.

☐ Ocasionalmente.

☐ Muy raramente.

☐ Nunca.

5. Respecto a mi relación con el bebé, en general:

☐ Me siento muy culpable por no implicarme más.

☐ Me siento bastante culpable por no implicarme más.

☐ Me siento moderadamente culpable por no implicarme más.

☐ Me siento ligeramente culpable por no implicarme más.

☐ No me siento nada culpable por no implicarme más.

6. Cuando estoy con el bebé, me aburro:

☐ Muy frecuentemente.

☐ Frecuentemente.

☐ Ocasionalmente.

☐ Muy raramente.

☐ Nunca.

7. Cuando estoy con el bebé y otras personas están presentes, me siento orgulloso de él:

☐ Muy frecuentemente.

☐ Frecuentemente.

☐ Ocasionalmente.

☐ Muy raramente.

☐ Nunca.

8. Procuro implicarme en el cuidado del bebé tanto como puedo:

☐ Muy frecuentemente.

☐ Frecuentemente.

☐ Ocasionalmente.

☐ Muy raramente.

☐ Nunca.

9. Me encuentro hablando a otras personas distintas a mi pareja sobre el bebé:

☐ Muy frecuentemente.

☐ Frecuentemente.

☐ Ocasionalmente.

☐ Muy raramente.

☐ Nunca.

10. Cuando no tengo más remedio que dejar el bebé:

☐ Me cuesta mucho irme.

☐ Me cuesta bastante irme.

☐ Me cuesta algo irme.

☐ Me cuesta poco irme.

☐ No me cuesta nada irme.

11. Cuando estoy con el bebé disfruto:

☐ Muy frecuentemente.

☐ Frecuentemente.

☐ Ocasionalmente.

☐ Muy raramente.

☐ Nunca.

12. Cuando no estoy con el bebé, me doy cuenta de que pienso en él:

☐ Casi todo el tiempo.

☐ Muy frecuentemente.

☐ Frecuentemente.

☐ Ocasionalmente.

☐ Nunca.

13. Cuando estoy con el bebé intento alargar el tiempo que estoy con él/ella:

☐ Muy frecuentemente.

☐ Frecuentemente.

☐ Ocasionalmente.

☐ Muy raramente.

☐ Nunca.

14. Cuando he estado un tiempo separado del bebé y estoy a punto de encontrarme con él/ella de nuevo, normalmente siento:

☐ Placer intenso ante la idea.

☐ Placer moderado ante la idea.

☐ Leve placer ante la idea.

☐ Ningún sentimiento ante la idea.

☐ Sentimientos negativos ante la idea.

15. En los últimos tres meses me he encontrado embobado mirando al bebé:

☐ Muy frecuentemente.

☐ Frecuentemente.

☐ Ocasionalmente.

☐ Muy raramente.

☐ Nunca.

16. En este momento pienso en el bebé como algo propio, como algo mío:

☐ Completamente como MI bebé.

☐ Bastante como MI bebé.

☐ Algo como MI bebé..

☐ Un poco como mi bebé.

☐ Aún no lo siento como MI bebé..

17. Respecto a las cosas que hemos tenido que dejar por causa del bebé:

☐ Me arrepiento completamente.

☐ Me arrepiento bastante.

☐ Me arrepiento algo.

☐ Me arrepiento poco.

☐ No me arrepiento para nada.

18. En los últimos 3 meses, he sentido que no tenía tiempo para mi mismo o para hacer las cosas que me interesan:

☐ Muy frecuentemente.

☐ Frecuentemente.

☐ Ocasionalmente.

☐ Muy raramente.

☐ Nunca.

19. Normalmente cuando estoy con el bebé:

☐ Soy muy impaciente.

☐ Soy bastante impaciente.

☐ Soy moderadamente impaciente.

☐ Soy ligeramente impaciente.

☐ No soy nada impaciente.
